# P-1258. Unlocking Potentials: The Impact of Meropenem, Meropenem-vaborbactam, and Ceftazidime-avibactam in Combatting Carbapenem-Resistant *Enterobacter cloacae* in Epithelial Lining Fluid and Serum

**DOI:** 10.1093/ofid/ofae631.1440

**Published:** 2025-01-29

**Authors:** Thomas Lavoie, Kathryn E Daffinee, jason M Pogue, Brahim Achour, Kerry L LaPlante

**Affiliations:** Infectious Diseases Research Program, Providence Veterans Affairs Medical Center, Providence, RI; College of Pharmacy, University of Rhode Island, Kingston, RI, mattapoisett, Massachusetts; Providence VA Medical Center, Providence, Rhode Island; University of Michigan, College of Pharmacy, Ann Arbor, MI; College of Pharmacy, University of Rhode Island, Kingston, RI, Kingston, Rhode Island; 1. Infectious Diseases Research Program, Providence Veterans Affairs Medical Center, Providence, RI, United States 2. Center of Innovation in Long-Term Support Services, Providence Veterans Affairs Medical Center, Providence, RI, United States 3. College of Pharmacy, University of Rhode Island, Kingston, RI, United States 5. Warren Alpert Medical School of Brown University, Division of Infectious Diseases, Providence, RI, Kingston, Rhode Island

## Abstract

**Background:**

Carbapenem-resistant Enterobacterales (CRE) are an urgent threat. We modeled epithelial lining fluid (ELF) and serum concentrations of meropenem (MER), meropenem-vaborbactam (MV), and ceftazidime-avibactam (CA) to assist in treatment selection.

Meropenem, meropenem-vaborbactam, and ceftazidime-avibactam dosing simulating ELF concentrations with a non-CP-producing CRE

**Figure:**
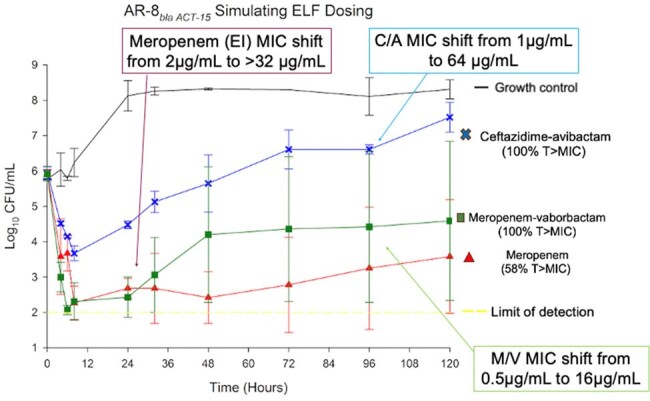

**Methods:**

Two *E. cloacae* were evaluated, one a non-carbapenemase (CP) producing isolate with multiple porin mutations and *bla_ACT-15,_* and one *bla_KPC-3_* CP-producing isolate. One compartment IVPD models were run in duplicate for ELF and humanized serum concentration targets. Models were sampled for CFU/mL counts at 0, 4, 6, 8, 24, 32, 48, 72, 96, and 120h. Concentrations were extrapolated from population-pharmacokinetic data to reflect free drug concentrations in ELF and serum. Antibiotics were administered as a bolus, with serum models also run as an extended infusion (EI). Susceptibility changes, via E-test, were evaluated every 24h and compared to 0h. Bactericidal activity was defined as ≥3log_10_ CFU/mL reduction from the initial inoculum, and bacteriostatic activity as < 3 log_10_ CFU/mL reduction.

Meropenem, meropenem-vaborbactam, and ceftazidime-avibactam dosing simulating serum concentrations at steady state with a CP-producing CRE

**Figure:**
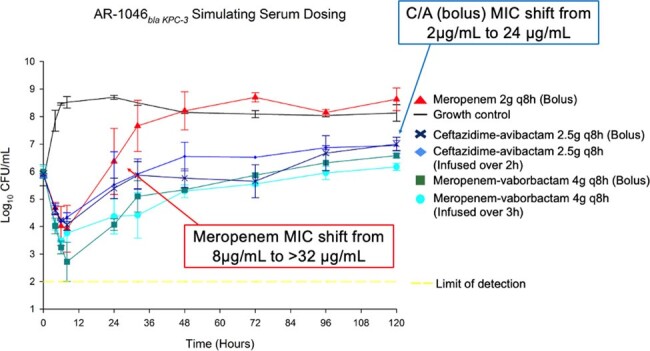

**Results:**

B*la_ACT-_*_15_ isolate: ELF concentrations of MER and MV were bactericidal through 24h with MIC shifts in both treatments, including MER MIC shifts to >32μg/mL and MV MIC shifts to 16μg/mL. CA ELF concentrations were bacteriostatic through 48h before bacterial regrowth above the initial inoculum and MIC shifts to 64μg/mL. All bolus serum antimicrobial concentrations resulted in bactericidal activity, however, only MV remained bactericidal at 120h with no MIC shifts. EI-MER and EI-MV alternatively were bacteriostatic after 48h.

B*la_KPC-_*_3_ isolate: Bactericidal activity was only observed with MV, in ELF models with no MIC shift. CA and MER ELF concentrations had bacterial regrowth after 24h. MIC shifts included a change to >256μg/mL after CA exposure. Serum concentrations of CA and MV were bacteriostatic through 24h. CA and MV serum models had bacterial regrowth after 72h with MIC shifts to 16μg/mL for CA. No MIC shifts were observed with serum MV. Bolus and EI results were similar for all.

Meropenem, meropenem-vaborbactam, and ceftazidime-avibactam dosing simulating serum concentrations at steady state with a non-CP-producing CRE

**Figure:**
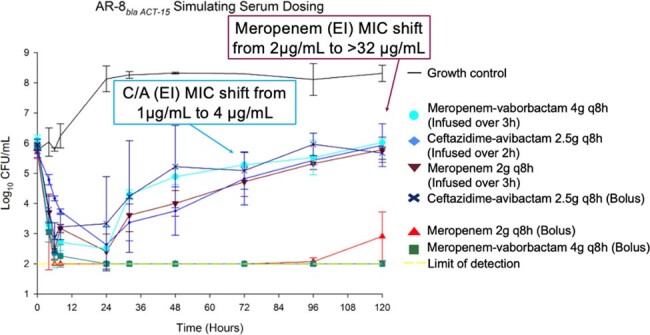

**Conclusion:**

MV serum concentrations were the most active therapy, with no MIC shifts, for two CRE *E. cloacae* IVPD models. ELF concentrations should be considered as a contributing factor to treatment failure and resistance development.

Meropenem, meropenem-vaborbactam, and ceftazidime-avibactam dosing simulating ELF concentrations with a CP-producing CRE

**Figure:**
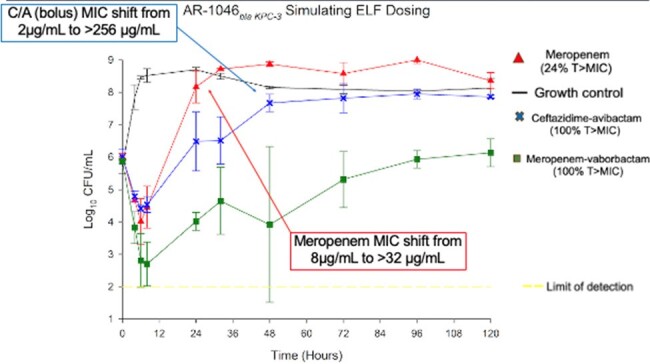

**Disclosures:**

**jason M. Pogue, PharmD**, Entasis: Advisor/Consultant|GSK: Advisor/Consultant|Melinta: Advisor/Consultant|Melinta: Grant/Research Support|Merck: Advisor/Consultant|Merck: Grant/Research Support|Shionogi: Advisor/Consultant|Shionogi: Grant/Research Support|Venatorx: Advisor/Consultant **Kerry L. LaPlante, Pharm.D., FCCP, FIDSA, FIDP**, Abbive: Advisor/Consultant|Abbive: Grant/Research Support|Ferring: Advisor/Consultant|Gilead: Grant/Research Support|Melinta: Advisor/Consultant|Merck: Grant/Research Support|Pfizer: Grant/Research Support

